# Familial aggregation and heritability of markers of metabolic risk, physical activity, and physical fitness in nuclear families from Muzambinho (Minas Gerais, Brazil)

**DOI:** 10.20945/2359-3997000000137

**Published:** 2019-04-26

**Authors:** João Paulo dos Anjos Souza Barbosa, Luciano Basso, Teresa Bartholomeu, Januária Andrea Souza Rezende, Jorge Alberto de Oliveira, António Prista, Go Tani, José António Ribeiro Maia, Cláudia Lúcia de Moraes Forjaz

**Affiliations:** 1 Universidade de São Paulo Laboratório de Hemodinâmica da Atividade Motora Escola de Educação Física e Esporte Universidade de São Paulo SP Brasil Laboratório de Hemodinâmica da Atividade Motora, Escola de Educação Física e Esporte, Universidade de São Paulo, SP, Brasil; 2 Universidade de São Paulo Laboratório de Comportamento Motor Escola de Educação Física e Esporte Universidade de São Paulo SP Brasil Laboratório de Comportamento Motor, Escola de Educação Física e Esporte, Universidade de São Paulo, SP, Brasil; 3 Instituto Federal do Sul de Minas Gerais Instituto Federal do Sul de Minas Muzambinho MG Brasil Instituto Federal do Sul de Minas – Campus Muzambinho, Muzambinho, MG, Brasil; 4 Centro de Investigação e Desenvolvimento do Desporto e Actividade Física Faculdade de Educação Física e Desporto Universidade Pedagógica Maputo Moçambique Núcleo de Investigação em Actividade Física e Saúde, Centro de Investigação e Desenvolvimento do Desporto e Actividade Física (CIDAF), Faculdade de Educação Física e Desporto (FEFD), Universidade Pedagógica, Maputo, Moçambique; 5 Universidade do Porto Centro de Investigação, Formação, Intervenção e Inovação em Desporto Faculdade de Desporto Universidade do Porto Porto Portugal Laboratório de Cineantropometria e Gabinete de Estatística Aplicada, Centro de Investigação, Formação, Intervenção e Inovação em Desporto (CIFI2D), Faculdade de Desporto, Universidade do Porto, Porto, Portugal

**Keywords:** Cardiovascular risk factors, motor activity, muscle strength, families, genetic epidemiology

## Abstract

**Objective:**

This study investigated the familial aggregation and heritability of markers of metabolic risk, physical activity, and physical fitness in nuclear families from Muzambinho (Minas Gerais, Brazil).

**Subjects and methods:**

The study included members of 139 families, comprising 97 fathers (aged 40 ± 7 years), 129 mothers (35 ± 6 years), 136 sons (12 ± 4 years), and 121 daughters (12 ± 5 years). Evaluated markers included (A) body mass index, waist circumference, glycemia, and cholesterolemia, as metabolic risk markers; (B) total weekly volume of physical activity, as a physical activity marker; and (C) relative muscle strength, as a physical fitness marker. Correlations between family members and heritability (h^2^) were estimated using the software S.A.G.E.

**Results:**

Significant familial correlations were obtained between parents-offspring for glycemia and cholesterolemia (both ρ = 0.21, p < 0.05) and relative muscle strength (ρ = 0.23, p < 0.05), and between siblings for waist circumference, glycemia, total weekly volume of physical activity, and relative muscle strength (ρ variation 0.25 to 0.36, p < 0.05). Heritability values were significant for almost all variables (h^2^ variations: 20% to 57% for metabolic risk markers, 22% for the total weekly volume of physical activity, and 50% for relative muscle strength), except for waist circumference (h^2^ = 15%, p = 0.059).

**Conclusion:**

The presence of significant correlations between family members and/or significant heritability strengthens the possible genetic and/or common familial environment influence on metabolic risk markers, total weekly volume of physical activity, and relative muscle strength.

## INTRODUCTION

Cardiovascular diseases are the main cause of death worldwide (1) and in Brazil (2). Several factors, like the presence of metabolic disease (*e.g.,* obesity, diabetes, and dyslipidemia), are known to increase cardiovascular risk (1). On the other hand, regular practice of physical activity is considered a cardiovascular protective factor. Physical fitness also has cardiovascular protective effects (3), and body strength has been recently shown to be inversely related to cardiovascular morbidity and mortality (4). Since all these factors may be affected by genetic and environmental influences, it is important to identify the magnitude of the contribution of each influence (genetic and environmental) to understand how different interventions may affect an individual’s cardiovascular risk.

Genetic epidemiology is a research area focused on the investigation of possible genetic influences on a trait (5,6). The first two steps of a genetic epidemiology study are the identification of significant familial aggregation (greater correlations of a trait within family members than expected by chance) and heritability (proportion of a trait variability in a population that may be explained by genetic factors) of the trait, which are assumptions necessary to ensure the presence of genetic influence (6,7). However, familial aggregation and heritability vary among different populations, since environmental influences differ with location and time, reducing or increasing the genetic influence (6-8). Familial aggregation and heritability of a trait have a specific value for each population; therefore, these characteristics must be studied in different contexts because the replication of significant values reinforces the genetic involvement in determining a specific trait.

Familial aggregations of markers of metabolic risk, physical activity, and physical fitness have been investigated outside Brazil. Studies from France, United States, and Portugal have found greater correlations between biological relatives (parents-offspring and siblings) than between father-mother for body mass index (BMI) (9), glycemia (10), and metabolic syndrome index (11), supporting a genetic influence on these traits. Regarding total physical activity, familial aggregation between genetically related relatives varies from an absence of correlation (12) to significant correlations (r = 0.34), as found in a study by Maia and cols. (13). For physical fitness, studies of familial aggregation are limited to aerobic fitness (14,15).

Similarly, the heritability of markers of metabolic risk, physical activity, and physical fitness has been studied in different countries and in a single study conducted in Brazil. As expected, heritability varies substantially in different populations. Systematic reviews have shown that heritability of BMI ranges from 30% to 76% (8). In the Brazilian study mentioned above, the heritability of metabolic risk markers varied from 29% to 51% (16). Regarding total physical activity, heritability varies from 10% to 30% (17) and was 35% in the Brazilian study (18). To the best of our knowledge, the heritability of muscle strength has not been investigated in Brazilian nuclear families.

Based on the exposed above, the study of familial aggregation and heritability is important to ensure the genetic influence in different contexts. It is also important to study these aspects in Brazil, where only a single study in this area has been performed to date. Thus, the objectives of this study were to investigate the familial aggregation and heritability of markers of metabolic risk (BMI, waist circumference, glycemia, and cholesterolemia), physical activity (weekly volume of total physical activity), and physical fitness (relative muscle strength) in nuclear families from the municipality of Muzambinho (Minas Gerais, Brazil). Our hypothesis was that all markers of metabolic risk, physical activity, and physical fitness would present significant familial aggregation and heritability.

## SUBJECTS AND METHODS

A sample of families was recruited among 10-year-old students participating in a large study about growth, maturation, and motor development in Muzambinho, (19). Data regarding blood pressure heritability from this study have already been published (20).

The study was approved by the Ethics Committee of the School of Physical Education and Sport at the University of São Paulo (Brazil), and written informed consent was obtained from all participants.

Data were collected between March 2008 and November 2009 in Muzambinho, a small city in the state of Minas Gerais, Brazil. At the time of data collection, the population of Muzambinho was 19,925 and included 5,650 residents aged 5 to 19 years. The municipality has a human developmental index of 0.801, and its main economy is based on agriculture, livestock, and handicrafts (21). The school system, at the time of data collection, consisted of 13 schools (2 private and 11 public schools, including 3 state and 8 municipal ones) with 2,996 school-age children enrolled (321 in the private schools, 1,845 in the state schools, and 830 in the municipal schools) (21). The city had 11 healthcare facilities at data collection. The number of deaths related to cardiovascular diseases was 35 in 2008 and 37 in 2009, representing 28% and 31%, respectively, of the deaths in the municipality each year (22).

The study investigators visited the selected families at their homes to explain the study’s procedures, obtain written consent for participation in the study, and schedule a new visit to the home the next morning. In this second visit, fasting blood samples were collected from all family members, and a third appointment was set for an evening visit at one of the city’s schools. In this third appointment, anthropometric measurements, physical activity habits, and physical fitness were assessed.

### Measurements

Weight (kg) and height (m) were measured with an electronic scale (Filizola, São Paulo, Brazil) following standard procedures, and the BMI was calculated as weight (kg)/height (m)^2^. Waist circumference was measured at the level of the umbilicus (23,24). Based on BMI values, the participants were classified as normal, overweight, or obese according to international guidelines (24,25). International tables were used to classify the participants’ waist circumference (23,24).

Glycemia and cholesterolemia were measured using automatic devices (Accu-Chek, Roche, São Paulo, Brazil; and Accutrend GC, Roche Diagnostics, Mannheim, Germany, respectively) after at least 6 hours of fasting (26,27).

Physical activity was assessed by a specific and culturally appropriate structured interview conducted with all family members together. The main questions included the family’s usual commuting physical activity (how they commute from one place to another in the city, if walking, cycling, etc.), occupational physical activity (what they do for work, if domestic chores, rural tasks, deliveries, etc.), and leisure time physical activity (what they do during leisure time, if cycling, soccer, swimming, gym classes, ballet, or physical play/games such as tag, hide-and-seek, hopscotch, skip rope, and others). In addition, children were asked about their physical activity at school (including physical education classes and physical activity during breaks). When the participant declared to perform physical activity in any of the mentioned domains (commuting, occupational, school, and/or leisure time), the weekly frequency and duration of the activity were questioned, and the weekly volume of the activity was calculated as the product of frequency and duration. The weekly volume of total physical activity (min/week) was obtained by the unweighted sum of the volumes of all physical activities performed.

Muscle strength was assessed by the isometric handgrip test using a hydraulic hand dynamometer (SH 5001, Saehan Corporation, Masan, South Korea). After adjustment of the handle of the equipment to the size of the volunteer’s hand, each volunteer performed a handgrip movement using the maximum strength possible with the dominant hand, and the value obtained was recorded (28). This value was relativized by body weight, yielding the relative muscle strength.

### Statistical analysis

Initial exploratory and descriptive analyses were performed using SPSS 20.0 (SPSS Inc., Chicago, IL, USA). Two-way analysis of variance (ANOVA) was used to compare traits between generations and sexes. The chi-square test was used to compare categorical variables between family groups. The description of the nuclear families was obtained with PEDSTATS (29). Analysis of missing values was carried out using SYSTAT 13 (Systat Software, Inc., San Jose, CA, USA) within each family structure, and missing data distribution was carried out at random. Correlations of each marker between family members were calculated using regression residuals after adjustment for the covariates age, sex, age X sex, age^2^, and age^2^ X sex, as suggested by Bouchard and cols. (30). Correlations between parent-offspring (ρ_PO_), siblings (ρ_SI_), and father-mother (ρ_FM_) were calculated using the software S.A.G.E. (31). Heritability (h^2^) was also estimated with S.A.G.E. (31) using Student’s *t* distribution for robust estimation of the parameters. This analysis was preceded by adjustments for the covariates age, sex, age X sex, age^2^, age^2^ X sex.

The level of significance was set at 5% for all analyses.

## RESULTS

In all, 210 families were invited to participate in the study. Data were effectively obtained from 139 families ranging in size from 3 to 9 members, with most families having 3 or 4 members (81%).

In some families, the mother and (particularly) the father were absent in the evaluation. Thus, the final sample consisted of 97 fathers, 129 mothers, and 257 children (136 sons and 121 daughters). The age of the offspring ranged from 6 to 24 years (sons 12 ± 4 years; daughters 12 ± 5 years), and the parents’ age ranged from 24 to 65 years (fathers 40 ± 7 years; mothers 35 ± 6 years). Concerning the socioeconomic status of the participants, only 10.8% of the families had at least one of the parents with an occupational activity requiring a university degree. Regarding family health history, 51.1% of the families reported a history of cardiovascular disease.

As expected, BMI and waist circumference values were significantly higher in parents compared with offspring, but were comparable between sexes within the same generation (Table 1). When the BMI was analyzed as a categorical parameter, the frequency of excess weight (overweight and obesity) was similar between men and women of the same generation, and higher in the first generation (fathers [59.5%] = mothers [50.0%] > sons [22.0%] = daughters [20.0%], p < 0.05). The frequency of altered waist circumference was lower in fathers (9.5%) compared with all other family groups and was greater in mothers (44.2%) compared with sons (24.6%) and daughters (31.9%), who presented similar frequencies of altered waist circumference. Glycemia was significantly higher in fathers compared with sons and mothers, while cholesterolemia was higher in both parents compared with offspring and in fathers compared with mothers (Table 1).

**Table 1:** Descriptive information on markers of metabolic risk, physical activity, and physical fitness among family members from Muzambinho (MG, Brazil)

	Fathers Mean ± SE (n)	Mothers Mean ± SE (n)	Sons Mean ± SE (n)	Daughters Mean ± SE (n)
**Metabolic risk markers**				
BMI (kg/m^2^)	25.8 ± 0.36 **(n = 84)**	25.6 ± 0.41 **(n = 120)**	18.4 ± 0.31* **(n = 127)**	18.8 ± 0.39* **(n = 115)**
WC (cm)	91 ± 0.98 **(n = 84)**	87 ± 1.00 **(n = 120)**	66 ± 0.97* **(n = 127)**	67 ± 1.11* **(n = 115)**
Glycemia (mg/dL)	96 ± 1.25 **(n = 92)**	89 ± 0.89^#^**(n = 125)**	86 ± 0.77* **(n = 134)**	84 ± 0.73 **(n = 119)**
Cholesterolemia (mg/dL)	192 ± 3.67 **(n = 71)**	177 ± 2.30^#^**(n = 91)**	163 ± 1.88* **(n = 72)**	170 ± 2.75* **(n = 64)**
**Physical activity marker**				
WVTPA (min/week)	2018 ± 99.44 **(n = 83)**	2044 ± 94.05 **(n = 119)**	1320 ± 85.70* **(n = 126)**	1030 ± 70.30* **(n = 109)**
**Physical fitness marker**				
Relative strength (kg)	0.59 ± 0.01 **(n = 83)**	0.46 ± 0.01# **(n = 120)**	0.55 ± 0.01* **(n = 123)**	0.47 ± 0.01*^#^**(n = 109)**

SE: standard error; BMI: body mass index; WC: waist circumference; WVTPA: weekly volume of total physical activity. * p < 0.05 compared with first-generation family member of same sex.

^#^ p < 0.05 compared with males of the same generation.

The weekly volume of total physical activity was higher in parents compared with offspring and was similar between sexes at both generations. The relative muscle strength was higher in the first versus second generation and greater in males than females (father > mother > sons > daughters).

Table 2 presents the results of familial correlations. In regard to metabolic risk markers, there was no significant correlation between family members in terms of BMI. However, significant correlations were observed in terms of waist circumference between siblings (ρ = 0.25), glycemia between all family pairs (parents-offspring, ρ = 0.21; siblings, ρ = 0.28; and father-mother, ρ = 0.36), and cholesterolemia between parents-offspring (ρ = 0.21). Regarding the physical activity marker, the weekly volume of total physical activity correlated significantly between siblings (ρ = 0.36) and father-mother (ρ = 0.42). The physical fitness markers, relative muscle strength, correlated significantly between parents-offspring (ρ = 0.23) and siblings (ρ = 0.35).

**Table 2:** Correlations between different pairs of family members regarding markers of metabolic risk, physical activity, and physical fitness in nuclear families from Muzambinho (MG, Brazil)

Variables	ρ_PO_ ± SE	n pairs	ρ_SI_ ± SE	n pairs	ρ_FM_ ± SE	n pairs
**Metabolic risk markers**						
BMI (kg/m^2^)	0.03 ± 0.06	343	0.17 ± 0.11	109	0.03 ± 0.12	65
WC (cm)	0.01 ± 0.06	343	0.25 ± 0.09^†^	109	0.15 ± 0.12	65
Glycemia (mg/dL)	0.21 ± 0.07^†^	379	0.28 ± 0.09^†^	135	0.36 ± 0.09^†^	78
Cholesterolemia (mg/dL)	0.21 ± 0.09^†^	170	0.14 ± 0.16	39	-0.15 ± 0.14	46
**Physical activity marker**						
WVTPA (min/week)	0.10 ± 0.07	331	0.36 ± 0.09^†^	104	0.42 ± 0.10^†^	66
**Physical fitness marker**						
Relative strength (kg)	0.23 ± 0.06^†^	337	0.35 ± 0.11^†^	107	0.16 ± 0.12	61

BMI: body mass index; WC: waist circumference; WVTPA: weekly volume of total physical activity; ρ_PO_: parents-offspring correlation; ρ_SI_: siblings correlation; ρ_FM_: father-mother correlation. ^†^ p < 0.05.

Heritability values for markers of metabolic risk, physical activity, and physical fitness were all significant, except for waist circumference, for which a trend toward significance was detected (Table 3). Heritability for metabolic markers ranged from 15% for waist circumference (not significant, p = 0.059) to 57% for cholesterolemia. The heritability value was 22% for the weekly volume of total physical activity and 50% for the relative muscle strength.

**Table 3:** Maximal heritability (h2 ± SE) of markers of metabolic risk, physical activity, and physical fitness in nuclear families from Muzambinho (MG, Brazil)

Variables	h^2^ ± SE	p value
**Metabolic risk markers**		
BMI (kg/m^2^)	0.20 ± 0.09	0.019
WC (cm)	0.15 ± 0.09	0.059
Glycemia (mg/dL)	0.38 ± 0.08	< 0.001
Cholesterolemia (mg/dL)	0.57 ± 0.11	< 0.001
**Physical activity marker**		
WVTPA (min/week)	0.22 ± 0.08	< 0.001
**Physical fitness marker**		
Relative strength (kg)	0.50 ± 0.09	< 0.001

SE: standard error; BMI: body mass index; WC: waist circumference; SBP: systolic blood pressure; DBP: diastolic blood pressure; WVTPA: weekly volume of total physical activity.

## DISCUSSION

The main findings of the present study are: (A) metabolic risk markers, except for BMI, present significant correlations between pairs of genetically linked family members. All metabolic risk markers but waist circumference presented low to moderately significant heritability; (B) weekly volume of total physical activity showed significant familial aggregation between siblings and father-mother, as well as significant low heritability; and (C) relative muscle strength presented significant familial aggregation between parents-offspring and siblings, as well as moderate significant heritability.

The understanding of the concepts of familial aggregation and heritability is important for the interpretation of studies involving these two concepts. The presence of significant correlations between family members suggests a genetic and/or environmental influence shared by family members (common environment) on the trait (32). When correlations are significant between genetically linked pairs (parents-offspring and siblings), a genetic influence is possible, whereas in significant correlations observed only between nongenetically linked relatives (father-mother), a common environmental influence is more likely. When heritability is estimated in nuclear families, as done in the present study, separation of genetic and common environmental influences is unfeasible; thus, what is estimated is the maximal genetic influence supposing there is no effect from the common environment (32).

In general, significant familial correlations and/or heritability are observed for all metabolic risk markers, suggesting an influence of genes and/or common environmental on these traits (9,11) and corroborating the generally accepted idea that genes and familial environment partially determine an individual’s metabolic risk (33). However, considering the obesity markers assessed in our study, BMI showed no familial correlation and low heritability, while waist circumference only presented a significant correlation between siblings and a trend toward significant heritability. Although genetic and common environment may have had a role in determining the obesity risk in this population, their influence was weak. Heritability values of obesity markers vary considerably in the literature, ranging from 30% to 76% in different reviews (8,34), but the results found in the present study (20% for BMI and 15% for waist circumference) are below the limits reported previously. An important determinant of obesity is an individual’s eating habit, which is greatly influenced by cultural traditions (8). However, the eating habits of different families in small cities like Muzambinho are probably very similar, which explains the lower heritability observed in such populations. Unfortunately, the eating habits of the participants in this study were not evaluated and should be included in future studies.

Regarding other metabolic risk markers, correlations for glycemia were significant for all family pairs, with the highest value observed between father-mother, who share no genes, suggesting an important influence of common environment on this trait (35). The heritability value of glycemia (38%) was significant and similar to the one found in another Brazilian study conducted in Baependi (33%) and to the highest value reported in the literature (39%) (16). Cholesterolemia showed a significant correlation among parents-siblings and a moderate heritability (57%), which was higher than both the value reported in the Baependi study (29%) and the highest value reported in the literature (51%) (36). Discrepancies in cholesterolemia heritability between Baependi and Muzambinho may be explained, at least in part, by different sampling strategies. The Baependi study involved many family generations, while the present study involved nuclear families with school-age offspring, who likely share the family environment more frequently among themselves than with distant family members like cousins, uncles, and grandparents. Together, these results suggest that a common environment may have a great influence on cholesterolemia. Based on previous discussion, it is possible to propose that interventions on unique environments and family common environments are more prone to have positive results in reducing obesity and other metabolic markers, respectively.

Weekly volume of total physical activity presented significant correlations between siblings (r = 0.36) and father-mother (r = 0.42), and a significant but low heritability of 22%. As significant correlations were observed between both genetically and nongenetically related relatives, and were higher between the nongenetically related pairs, these results suggest that a common environment may influence this trait (32). The fact that the correlations were identified for pairs within the same generation (siblings and father-mother) suggests a special influence of the common environment shared by members of each generation in determining the weekly volume of physical activity (37). The low heritability value found for this trait is within the range reported in a review (6-62%) (38) and similar to the values found by Chaves and cols. (24%) (39), but lower than those reported in the Baependi study (35%) (18). This low heritability suggests that unique environments may have an important influence in determining this trait. Considering together the results from the familial aggregation and heritability, interventions aiming to increase the weekly volume of physical activity in this population should focus in the common environment shared by relatives from the same generation and the extra-familial environment.

To the best of our knowledge, this is the first study to investigate the familial aggregation and heritability of relative muscle strength in Brazilian nuclear families. Relative muscle strength had a significant correlation between parents-offspring and siblings and a moderate heritability (h^2^ = 0.50). These results suggest that half of the variation of relative muscle strength in Muzambinho’s population might be explained by genetic factors and/or a common familial environment. This result is aligned and within the range of isometric strength shown in other international studies involving nuclear families, showing estimates of heritability involving this type of strength of 27% to 58% (40).

Despite the importance of these findings, this study has some limitations. The sample derived from a population with high levels of weekly total physical activity. Also, due to the selection criteria (families of 10-year-old children), the sample consisted of families with young adults as parents. These features may reduce the chance of metabolic risk abnormalities and increase the influence of the common familial environment. Since we had no previous knowledge of the physical activity patterns of this population at the time of data collection, we applied a structured open interview (20,41). However, interviews are vulnerable to some degree of inaccuracy; thus future studies should implement measures that are more objective. The size of the cohort of the present study may be considered insufficiently large, but is similar to that of previous studies (19,20,41) and represented 2.2% of the population of Muzambinho.

In conclusion, in families from Muzambinho, markers of metabolic risk, weekly volume of total physical activity, and manual strength showed significant familial aggregation and/or heritability, suggesting a genetic and common environmental influence on these traits.
